# Using data from the Microsoft Kinect 2 to determine postural stability in healthy subjects: A feasibility trial

**DOI:** 10.1371/journal.pone.0170890

**Published:** 2017-02-14

**Authors:** Behdad Dehbandi, Alexandre Barachant, Anna H. Smeragliuolo, John Davis Long, Silverio Joseph Bumanlag, Victor He, Anna Lampe, David Putrino

**Affiliations:** 1 Department of Telemedicine and Virtual Rehabilitation, Burke Medical Research Institute, White Plains, New York, United States of America; 2 Department of Rehabilitation Medicine, Weill-Cornell Medical College, New York, New York, United States of America; 3 Clinical Laboratory for Early Brain Injury Recovery, Burke Medical Research Institute, White Plains, New York, United States of America; 4 Langone School of Medicine, New York University, New York, New York, United States of America; 5 Department of Physical Therapy, Mercy College, Dobbs Ferry, New York, United States of America; Purdue University, UNITED STATES

## Abstract

The objective of this study was to determine whether kinematic data collected by the Microsoft Kinect 2 (MK2) could be used to quantify postural stability in healthy subjects. Twelve subjects were recruited for the project, and were instructed to perform a sequence of simple postural stability tasks. The movement sequence was performed as subjects were seated on top of a force platform, and the MK2 was positioned in front of them. This sequence of tasks was performed by each subject under three different postural conditions: “both feet on the ground” (1), “One foot off the ground” (2), and “both feet off the ground” (3). We compared force platform and MK2 data to quantify the degree to which the MK2 was returning reliable data across subjects. We then applied a novel machine-learning paradigm to the MK2 data in order to determine the extent to which data from the MK2 could be used to reliably classify different postural conditions. Our initial comparison of force plate and MK2 data showed a strong agreement between the two devices, with strong Pearson correlations between the trunk centroids “Spine_Mid” (0.85 ± 0.06), “Neck” (0.86 ± 0.07) and “Head” (0.87 ± 0.07), and the center of pressure centroid inferred by the force platform. Mean accuracy for the machine learning classifier from MK2 was 97.0%, with a specific classification accuracy breakdown of 90.9%, 100%, and 100% for conditions 1 through 3, respectively. Mean accuracy for the machine learning classifier derived from the force platform data was lower at 84.4%. We conclude that data from the MK2 has sufficient information content to allow us to classify sequences of tasks being performed under different levels of postural stability. Future studies will focus on validating this protocol on large populations of individuals with actual balance impairments in order to create a toolkit that is clinically validated and available to the medical community.

## Introduction

It is estimated that each year, fall-related injury costs the United States $34 billion in direct costs [1]. For elderly individuals alone, fall-related visits to the emergency department have been estimated to cost an average of $2,823 per visit, with each fall-related hospitalization estimated to cost an average of $25,465 [1]. Methods for stratifying fall risk and focusing preventive measures on those who are at highest risk remains one of the most important management strategies for saving resources and preventing suffering [1–3]. However, sensitive and reliable measures for assessing balance and fall-risk are difficult to develop and implement. One widely used balance measure, the Berg Balance Scale (BBS), relies on subjective assessment, and most of its domains require the ability to stand, thereby limiting scoring range and applicability toward non-ambulatory subjects [3–5]. By contrast, computerized posturography is highly sensitive, but requires costly specialized equipment only found in institutional settings [6–8].

There are an unprecedented number of low cost electronic devices entering the consumer market that are capable of tracking metrics related to human movement [9]. However, despite the potential in this space for healthcare applications, these devices are yet to make their way into clinical practice. One such device, the Microsoft Kinect 2 (MK2; Microsoft Corporation, Redmond, WA) represents significant innovation in the field of accessible and affordable markerless motion capture. The MK2 features a 1080p (for HD video acquisition) camera, an active infrared (IR) camera, and a depth sensor (for 3-Dimensional (3D) skeletal tracking). The resultant technology has the ability to perform 3D tracking of head, trunk, limb, and finger motion by tracking the location of 25 inferred joint centroids across the entire body. The MK2 has a well-developed Application Programming Interface (API) to support the development of custom applications that utilize the raw data collected by the MK2. This innovative technology represents a unique opportunity to create low-cost, automated software systems that can quantify motor behavior and impairment.

The MK2 (and many devices like it) are already being used for human activity recognition, and often utilize state-of-the-art machine learning paradigms to perform their intended roles [10–13]. Furthermore, the MK2 and Kinect 1 have both been used in the past to make balance assessments using a variety of different techniques [14–18]. However, the existing Machine-learning approaches for the analysis of Kinect data are designed to solve the problem of gesture classification, such as the work described by Li and colleagues [19]. The major focus of this work is to solve a slightly different problem: to classify differences in the relative “quality” of the same gesture being performed in different ways. This is a more challenging problem, and conventional approaches that have previously been used to analyze MK2 data have not addressed this unmet need. The purpose of this study is to investigate the ability of a novel machine learning approach to classify the relative movement quality of different gestures. We hope that this will permit us to make automated determinations about balance ability in our subjects.

There is a critical need for low-cost technology that can quantify balance ability in individuals in a way that is fast, simple, and clinically relevant. It is important that these systems be accessible enough to be rapidly deployed to home environments so that individuals who fall into at-risk populations are capable of safely testing balance at home, rather than facing the financially and logistically challenging prospect of regular clinical check-ups. Here, we propose a modified BBS (mBBS) assessment that can be performed from a seated position. We chose a seated assessment position because it would allow us to test non-ambulatory subjects, and it will allow subjects to perform the assessment safely in an unsupervised environment. We then assess the feasibility of using the MK2 as a tool for balance assessment by comparing data output from the MK2 to data collected from a research-grade force platform. Finally, we investigate whether the data collected from the MK2 can be used to identify differing levels of postural stability during performance of balance assessment tasks.

## Materials and methods

### Data collection

#### Subjects

Twelve healthy subjects were recruited and data was collected and analyzed with the full approval of the Burke Rehabilitation Hospital Committee for Human Rights in Research (protocol approval number: BRC509). Subjects were recruited from the staff at the Burke Medical Research Institute if they had no history of neurological disorder or serious musculoskeletal injury, displayed healthy upper and lower limb function and were under the age of 65. Because all subjects were healthy individuals engaging in a minimal risk protocol that was designed as a hardware testing protocol for the MK2, the Burke Rehabilitation Hospital Committee for Human Rights in Research deemed it appropriate for data collection to occur without the need for written consent. Verbal consent was obtained from all research participants prior to data collection, and consent was indicated on a Microsoft Excel Spreadsheet checklist. The subject population for this project included 8 females and 4 males (mean age 30.2 ± 6.0 years).

#### Hardware configuration and experimental setup

**Fig 1:**
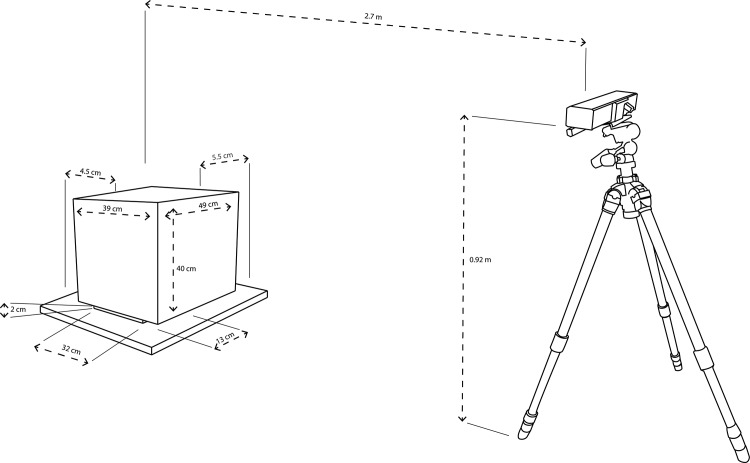
Experimental layout for data collection: subjects sat on a rectangular box that was placed in the center of the KFP at a distance of 2.7 meters from the MK2. The MK2 was mounted on a tripod 0.92 meters from the floor.

Data was collected using an MK2 that was connected to a Microsoft Surface Pro 3 tablet. In order to track joint centroids in real-time with the MK2, subject positioning was of paramount importance. For optimized tracking, subjects were positioned in front of the MK2 sensor that was placed (using a tripod) at a distance of approximately 2.7 meters from the center of the force plate, and 1.15 meters from the floor (Fig 1). This is within the optimal tracking range of sensors that are similar in nature to that of the MK2 [20,21]. The MK2 has a well-documented API that allows users to access much of the raw data that the device collects. For this project, we developed a custom data collection software program in Microsoft Visual Studio that harnessed the video and kinematic data-recording capabilities using the “Body Basics” and “Color Basics” functionalities of the MK2 API. The “Body Basics” system is optimized to skeletally track users while either standing or sitting, whereas “Color Basics” provides a video record of each data collection recording [22–24].

In order to assess the reliability of data recorded from the MK2, a Kistler Type 9260AA force plate (KFP) was used as a research-grade comparison. The KFP uses piezoelectric force sensors to accurately measure any forces applied to the surface of the device [25]. The intention of this study was to assess seated balance using the KFP and the MK2. However, the KFP was found to be very sensitive to the type of chair used due to the varying force transfer properties of different materials. In order to create experimental conditions that would ensure stable readings from the force platform, we designed and built a wooden stool (39cm x 49cm x 40cm) for subjects to sit on when completing the protocol. The weight of the subject was transferred to the KFP via two foot-shaped wooden blocks on the base of the box (32cm x 13cm x 2cm) in order to emulate a standing subject while facilitating weight transfer through the left and right ischial tuberosities of each subject. Raw data from the KFP is used to automatically calculate center of pressure (COP) data for each subject, using methods that are detailed completely in online Kistler resources that can be freely accessed at the following urls: http://bit.ly/2bnL9Lz (Overview of formulae used to derive metrics from the KFP raw data), http://bit.ly/2cjsOmM (general platform specifications). COP metrics from force platforms have been routinely used to evaluate postural stability in a number of studies, and have been shown to correlate moderately with other established measures of balance ability in both healthy and clinical populations [26–29].

The wooden box was placed in the center of the force plate. The force plate was positioned against a wall and the MK2 was set up on a tripod 2.7 meters from the center of the box. Prior to each data collection session, each subject sat in the center of the wooden box with both feet on the ground while the force plate calibrated and measured the subject’s seated weight. An experimenter also recorded each subject’s seated height (from the surface of the force plate to the tops of their heads). It was necessary to calibrate the force plate with a seated subject because subjects remained sitting for the entirety of the data acquisition phase.

#### Protocol and data acquisition

**Fig 2:**
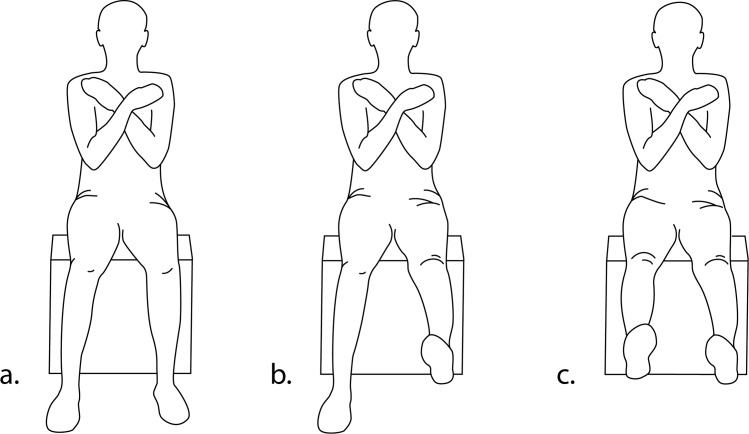
Three postural conditions. a) Both feet on the ground. b) One foot off the ground. c) Both feet off the ground.

For this project, each subject was asked to perform a modified version of the Berg Balance Scale (BBS). The BBS is a widely used, gold-standard metric for predicting risk of falling in multiple clinical and non-clinical populations [2,4]. The BBS involves fourteen physical tasks of varying complexity and difficulty that are designed to test an individual’s standing balance. However, our intention for this study was to develop a protocol that can test balance ability in individuals who are non-ambulatory, or give ambulatory individuals the ability to safely assess balance from a seated position. In order to test balance in the seated position, we developed a specialized “modified Berg Balance Scale” (mBBS) with six tasks that are closely related to the original BBS items, and also adapted from other work in this field (Table 1; [6]). Each subject was instructed to complete the six tasks of the mBBS protocol under three different conditions (Fig 2) that represented increasing challenges to postural stability:

1. Both feet on the ground
2. One foot off the ground (knee fully extended)
3. Both feet off the ground (both knees fully extended)

**Table 1:** Modified Berg Balance Scale tasks.

Modified Berg Balance Scale	
Task	Instruction
Task 1	With arms crossed, sit unsupported for 10 seconds.
Task 2	With arms crossed and eyes closed, sit unsupported for 10 seconds.
Task 3	Arms at your sides, lean back against the wall for 10 seconds. Without using your hands, sit up unsupported for 10 seconds. Repeat once.
Task 4	Arms crossed, look over your left shoulder for 10 seconds. Face front then turn to look over your right shoulder for 10 seconds. Return to face front.
Task 5	Pick up an object from the floor with your left hand, and return to sit upright, unsupported. Lean over to place the object back on the floor. Repeat using right hand. End in upright seated position.
Task 6	Start with arms crossed, sitting unsupported. Raise one arm 90° out from your side. Hold for 10 seconds, then return to start position for 10 seconds. Repeat with other arm.

#### Comparison of MK2 and KFP data

The goal of this study is to determine whether the MK2 could be reliably used to assess postural stability with comparable accuracy a research-grade measurement tool (the KFP). Data from the two devices are of similar natures (multivariate time-series) and therefore can be handled using the same data processing pipeline. The comparison will be done on 3 levels:

1. Benchmarking the MK2. First we must ensure the quality of the data coming from the MK2. Observing the Pearson’s correlation and root mean squared error (RMSE) value between the signals from the two devices will act as a measure of the similarity between time-series.
2. Classification across tasks. In this setup, we are interested in predicting the task of a given trial. This is a gesture recognition problem where the MK2 is particularly adapted.
3. Classification across conditions. In this setup, we are looking to predict the postural condition under which the 6 tasks were performed. While the KFP is supposed to perform relatively well at this task, the ability of the MK2 to discriminate between the conditions remains the main question of this study.

#### Structure of raw motion capture data (MK2)

**Fig 3:**
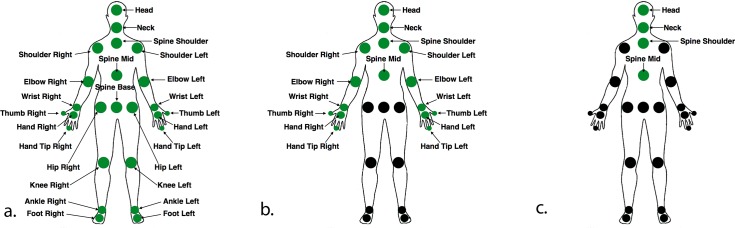
a) The 25 joint centroids that are used by the MK2 to characterize full body motion. b) The 16 joint centroids used to characterize the trunk and upper extremities. c) The four joint centroids used to characterize the spine.

The MK2 quantifies body motion by tracking the coordinates of 25 anatomical joint centroids in 3-dimensional space (Fig 3A). It records at a sampling frequency of 30Hz. Since variation in balance was emulated by asking the subject to perform the task under different leg postures (Fig 2), we were concerned that using data collected from the lower body joint centroids would lead to classification based on the posture itself, rather than postural stability. Therefore, for the purposes of this study, we only used 16 out of the 25 joint centroids that characterized motion of the upper part of the body: starting from the Spine Mid centroid and above (Fig 3B). We also chose to independently examine the four centroids that make up the spinal column: Spine Mid, Spine Shoulder, Neck, and Head (Fig 3C).

Positional X, Y, and Z coordinates were recorded for each of our 16 centroids. Let **x**_***MK*2**_(t) ∈ ℝ^48^ be a data recorded at the time-point t, we have:

$$x_{MK2}(t)=[J1_{x}(t),J1_{y}(t),J1_{z}(t),\ldots ,J16_{x}(t),J16_{y}(t),J16_{z}(t)]$$ (1)

Where (*J*1_*x*_(*t*),*J*1_*y*_(*t*),*J*1_*z*_(*t*)) are the x, y, z coordinates of the joint #1 (“Spine_Mid”) of the MK2.

As each subject was directed to perform the mBBS, a new data file was recorded for each of the 6 tasks of the mBBS, and data files were sorted according to task and condition. Therefore, each task of index *i* ∈ {1: *K*} is represented as a matrix *X*_*MK*2,*i*_ ∈ ℝ^48×*Nt*^ such as:

$$X_{MK2,i}=[{x_{MK2}(1)}^{T},\ldots ,{x_{MK2}(Nt)}^{T}]$$ (2)

where *Nt* is the number of frames in each recording and T is the transpose operator.

#### Structure of the raw force platform data (KFP)

The KFP provides 2-dimensional coordinates of the subject’s center of pressure in the horizontal plane. The sampling frequency is 600 Hz. Therefore, data from the KFP can be expressed in the same way as the MK2 data. Let ***x***_***KFP***_(***t***) denote the two dimensional time series recorded at the time-point t by the KFP, we have:

$$x_{KFP}(t)=[{COP}_{x}(t),{COP}_{y}(t)]$$ (3)

where (*COP*_*x*_(*t*),*COP*_*y*_(*t*)) are the x, y coordinates of the center of pressure of the subject. Following the same notation as in Eq (2), we can express the data corresponding to the task of index i by a matrix *X*_*KFP*,*i*_ ∈ ℝ^2×*Nt*^ such as:

$$X_{KFP,i}=[{x_{KFP}(1)}^{T},\ldots ,{x_{KFP}(Nt)}^{T}]$$ (4)

### Data analysis—Classification protocol

#### Covariance matrices and feature vectors

Covariance matrices are essential elements in the majority of algorithms and methodologies dedicated to the analysis of multivariate time series. They define all the second-order statistics of the data, which in regards to our two devices, can be seen as a measure of quantity and amplitude of movement. Specifically, in the context of the MK2 data, each task performed by the subject produces distinctive covariance structures, due to the constraints that link the joints of the skeleton. In addition, covariance estimation produces matrices of equal shape regardless the duration of the task, which varies from a subject to another. Covariance matrices estimation is also unaffected by irregular sample rate, which can be observed on low cost motion capture device such as the MK2. Finally, since postural instability is associated with compensatory movements, we expect to see changes in the covariance structure of the signal when the subject’s balance is challenged (as is the case in our three postural conditions). Lastly, the spatial covariance estimation (see Eq 5), by removing the first order moment of the signal, will be independent of the initial posture of the subject, insuring that the classification results will not be biased by this information. Therefore, we selected covariance matrices as the most appropriate and informative way to represent the kinematic features of the raw data originating from the MK2 and KFP. These matrices will be used as input of the classification pipeline. This approach has been successfully used to categorize human activity with a high degree of accuracy in recent works [30,31]. For the sake of simplicity, in the following equations we will use *X*_*i*_ for denoting a trial of a given task, regardless the acquisition device. Let *C*_*i*_ ∈ ℝ^*N*×*N*^ denote the covariance matrix of the trial *X*_*i*_ and N the number of time series of the corresponding device, the covariance is estimated by:

$$C_{i}=\frac{1}{Nt}{(X}_{i}-\bar{X_{i}}){{(X}_{i}-\bar{X_{i}})}^{T},$$ (5)

where T is the transpose operator and $\bar{X_{i}}$ the average across the Nt time samples of the time series.

Covariance matrices are symmetric and positive definite (SPD) matrices. Their manipulation must take this property into account in order to avoid loss or distortion of meaningful information embedded in the covariance structure. Riemannian geometry is a branch of differential geometry that studies Riemannian manifolds and can be used to manipulate SPD matrices [32]. In this framework, SPD matrices are seen as points of a Riemannian manifold with a canonical metric that allows for their manipulation while keeping their inner structure intact. In the past decade, this approach has been successfully used in numerous fields, such as EEG signal processing for Brain Computer Interface applications [33,34], radar signal processing [35], or image processing for pedestrian detection from video data [36]. Given two covariance matrices *C*_1_ and *C*_2_, the Riemannian distance is defined by [37]:

$$\delta_{R}(C_{1},C_{2})={\left[\sum_{i=1}^{N}{log}^{2}(\lambda_{i})\right]}^{1/2},$$ (6)

where *λ*_*i*_ are the real eigenvalues of $C_{1}^{-1}C_{2}$. This metric is invariant by affine transformation of the data, i.e. the distance between two covariance matrices is unaffected by any transformation that can be represented by an invertible matrix *V* ∈ ℝ^*N*×*N*^:

$$\delta_{R}(C_{1},C_{2})=\delta_{R}({VC}_{1}V^{T},VC_{2}V^{T}).$$ (7)

This includes any scaling or rotation of the data. In the context of MK2 data, this property makes the classification robust to small variations in starting position of the MK2 with respect to subject location, as well as differences in height and body structure of different subjects.

While Riemannian metrics have not previously been used for gesture recognition, other covariance-based distances (euclidean or log-euclidean) have been successfully used to classify different types of gestures with a nearest neighbor’s approach [30]. Performance of distance-based classifiers is highly dependent on the sensitivity of the metric used to measure similarity between points. With respect to other metrics, the Riemannian metric is more suited to measure differences between covariance matrices. However, the distance defined in Eq (6) integrates both noise and signal equally, and therefore can lack sensitivity in the most difficult cases i.e. when the classes are overlapping in most of the dimensions of the feature space. Our approach aims to classify variations in quality of the same gesture. As a consequence, classes are less separable, and distance-based classifiers failed to produce consistent and accurate results. To overcome this limitation, we relied on tangent space mapping. Tangent space mapping is a local projection that maps the elements of the manifold into an Euclidean space while keeping their distance relationships intact. Therefore, once projected into the tangent space, covariance matrices can be vectorized and fed into any state-of-the-art classifier, giving the algorithm the opportunity to produce a more complex decision function and in doing so, separate signal from noise. The use of tangent space for classification has been first introduced for image classification in [36], and showed superior accuracy over distance-based classification in other contexts [33,34]. A tangent space is defined locally (i.e. around a reference point that sets the origin of the tangent space). Let *S*_*i*_ denote a square symmetric matrix, projecting the covariance matrix *C*_*i*_ into the tangent space, we have:

$$S_{i}=Log(C^{-1/2}S_{i}C^{-1/2}),$$ (8)

where C is a covariance matrix called “reference point,” and Log(.) is the matrix logarithm. For any given point of the tangent space, we have equivalence between its Euclidean distance (Frobenius norm) and the Riemannian distance of the corresponding point to the reference point in the original manifold:

$$\delta_{R}(C_{1},C_{2})={\left\|S_{i}\right\|}_{F},$$ (9)

Therefore, the matrix *S*_*i*_ represents the position, with respect to the reference point, of the corresponding covariance matrix in the Riemannian manifold. The choice of the reference point can be seen as a parameter of the algorithm and can influence, although moderately, performance of the classification. In previous works [33,34,36], the Riemannian mean [32,38] of all the training data has been used as reference point for the tangent space mapping. In this work, we will use the log-euclidean mean as a computationally effective approximation of the Riemannian mean [39]:

$$C=Exp(\frac{1}{K}\sum_{i=1}^{K}Log(C_{i})).$$ (10)

The matrix *S*_*i*_, being a square symmetric matrix with N*(N+1)/2 unique elements, can be reduced and vectorized in order to produce a vector that can be fed into a standard classification algorithm. Let *s*_*i*_ ∈ ℝ^N*(*N*+1)/2^ denotes such a vector, we have:

$$s_{i}=upper(S_{i})$$ (11)

where the upper(.) operator keeps the upper triangular part of a symmetric matrix and vectorizes it by applying unity weight for diagonal elements and √2 weight for out-of-diagonal elements [36]. The purpose of this weight is to keep the norm equivalence between the matrix and its vectorized representation.

Algorithm 1 summarizes the process by which feature vectors can be generated from raw data. An implementation of this algorithm is available online as part of a Python (Python Software Foundation, https://www.python.org) toolbox called ‘*pyRiemann’ (**https*:*//github*.*com/alexandrebarachant/pyRiemann**)*. In addition, the full dataset and code utilized in this manuscript can be accessed on Dryad (doi:10.5061/dryad.fk4cb) and Github (https://github.com/alexandrebarachant/WMFT_JBHI).

Algorithm 1: Feature vectors estimation

Input: a set of K MK2 data matrix *X*_*i*_

Output: a set of K tangent vectors *s*_*i*_

    1.    Estimate covariance matrices *C*_*i*_—Eq (5)

    2.    Compute reference point *C*—Eq (10)

    3.    Compute tangent matrices *S*_*i*_–Eq (8)

    4.    Vectorize *S*_*i*_ to get the tangent vectors *s*_*i*_–Eq (11)

    5.    Return *s*_*i*_

#### Classification across tasks

**Fig 4:**
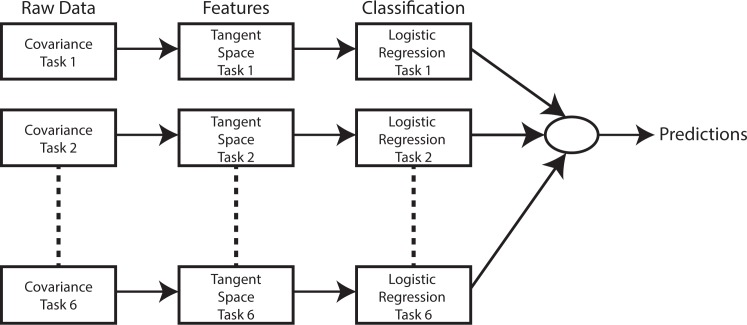
Classification pipeline for the classification across tasks.

Predicting which task the user is performing is a typical gesture recognition problem. In this setup, classes (i.e. the 6 tasks) are well separated in their feature representation domain. In this context, feature vectors will be directly classified by a simple Logistic Regression, regardless of the condition under which they have been recorded, as illustrated in Fig 4.

#### Classification across conditions

Classification across condition represents a more challenging problem. First, differences between conditions are of a smaller order of magnitude than differences between tasks, and therefore, classes are less separable. Second, the presence of six different tasks creates clusters in which each of the three conditions are nested. Therefore, it is very difficult to find a single model to separate each condition regardless of the task performed. In this context, we will train a classification model for each of the six tasks independently, and will rely on ensembling techniques in order to combine outputs of these six models into one single prediction. This pipeline is consists in applying the pipeline described Fig 4 independently on the data from each task. Ensembling of the six models is simply achieved by summation of the output probabilities of each individual logistic regression. Thus, the final prediction is given by the condition with the highest sum of probabilities.

## Results

### Study participants

Twelve subjects were recruited for the study and all subjects successfully completed the data collection protocol. We obtained 216 simultaneous recordings from both the KFP and the MK2 of each subject completing the 6 tasks of the mBBS under the three different postural conditions. The performance of each task was carefully timed, in order to ensure no differences arose due to timing discrepancies between subjects. Each task typically took no longer than 10 seconds (300 frames) to complete.

### Benchmarking KFP data against the MK2

**Fig 5:**
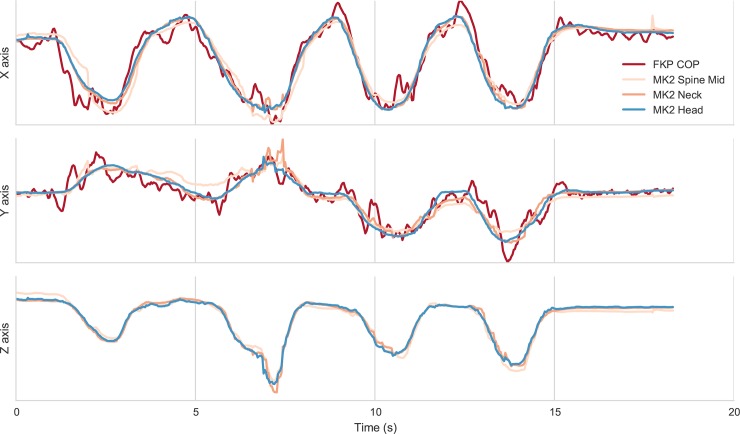
Visualization of raw data that was recorded during one subject’s performance of mBBS Task 5. We have plotted motion data from the KFP, the “Spine Mid” MK2 centroid, the “Neck” MK2 centroid and the “Head” MK2 centroid on the three axis. Note similarities in “x” and “y” directional data from the KFP and MK2 centroids. Each time series is normalized by its standard deviation for comparison.

Our first goal was to benchmark the data collected by the MK2 against that collected by the KFP in order to determine the quality of MK2 data as compared to a well-validated research-grade measuring device. Raw data was collected from both devices, and saved as.csv (KFP) and.bin (MK2) files that could be easily loaded into MATLAB (Mathworks, Natick MA) and Python for visualization (Fig 5). The first step in evaluating data from the MK2 in relation to a research-grade technology was to compare the variance of the COP metric reported by the KFP with the variance of the spinal centroid data recorded from the MK2.

**Fig 6:**
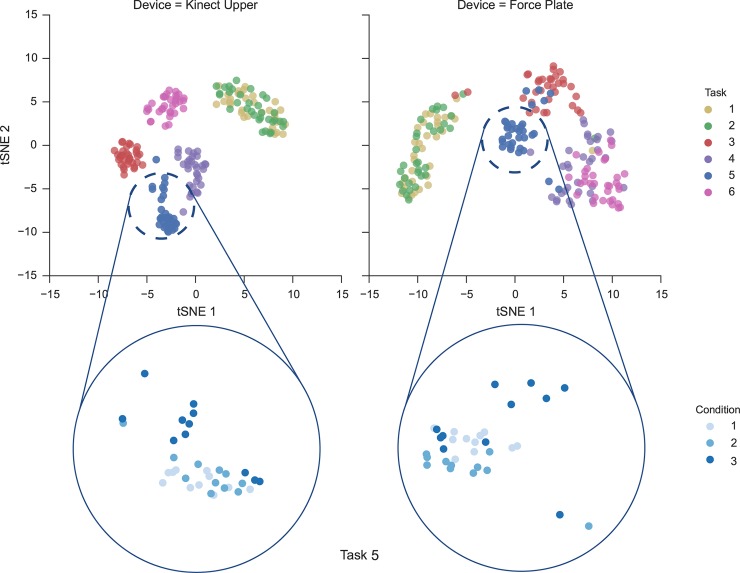
t-SNE representation of the dataset for the MK2 (left) and the KFP (right). A clear separation between tasks can be observed for both devices, but the MK2 data are more separable. Each cluster is formed by the points of the 3 conditions performed under the corresponding task. A zoom over the cluster of task 5 highlights a lower separation of the conditions.

Using t-SNE dimensionality reduction [36] in combination of the Riemannian metric, we are able to visualize our high dimensional dataset in a comprehensive fashion and have a first glance at the quality of the information being recorded by both devices (Fig 6). As seen in Fig 6, data form clusters that correspond to the different tasks. With the exception of the task 1 and 2: the subject being seated with eyes open (task 1) and eyes closed (task 2), all other tasks clearly lie in different regions of the Riemannian manifold. Given that we were working with healthy subjects, it should be stated that we did not expect to see a significant difference between data collected during task 1 and task 2. Despite tracking less information about body motion than the MK2, the force plate data are clearly separable. However, for tasks 3–6 the MK2 can actually better distinguish which task is being performed than the force plate. Additionally, for each cluster, we can find the difference between each of the postural 3 conditions. While the class separation ability is of a lower degree of magnitude, we can identify trends that can be attributed to change in postural stability.

In order to perform an initial, cursory examination of the similarities of the feature space between data from the KFP and MK2, we ran Pearson correlations between the variances in KFP COP data and the MK2 spinal centroid data. The highest correlations were seen in the Spine Mid, Neck, and Head centroids. The correlations for each task and both movement directions of KFP data were averaged across all subjects and presented in Table 2. All correlation coefficients were significant, with a p-value of less than 0.0001. The strong correlations observed between the variance in KFP and MK2 data output indicated to us that the MK2 data was of sufficient accuracy and sensitivity to be used for reliable classification analysis.

**Table 2:** Mean (± standard deviation) Pearson Rhos (r) showing the degree of variance correlation between selected spinal MK2 centroids and the KFP COP across all tasks. “X” and “Y” directions were named in reference to data coordinate system used by the KFP.

	Head (X)	Head (Y)	Neck (X)	Neck (Y)	Spine Mid (X)	Spine Mid (Y)
**Task 1**	0.91±0.08	0.89±0.06	0.94±0.07	0.92±0.06	0.91±0.08	0.87±0.07
**Task 2**	0.9±0.08	0.79±0.06	0.95±0.07	0.78±0.06	0.92±0.08	0.79±0.08
**Task 3**	0.78±0.08	0.88±0.06	0.88±0.07	0.76±0.06	0.65±0.08	0.86±0.08
**Task 4**	0.89±0.08	0.86±0.06	0.89±0.07	0.8±0.06	0.8±0.08	0.9±0.08
**Task 5**	0.87±0.08	0.9±0.06	0.8±0.07	0.95±0.06	0.84±0.08	0.88±0.08
**Task 6**	0.72±0.08	0.67±0.06	0.79±0.07	0.92±0.06	0.89±0.08	0.8±0.08

### Classification of conditions from MK2 and KFP data

**Fig 7:**
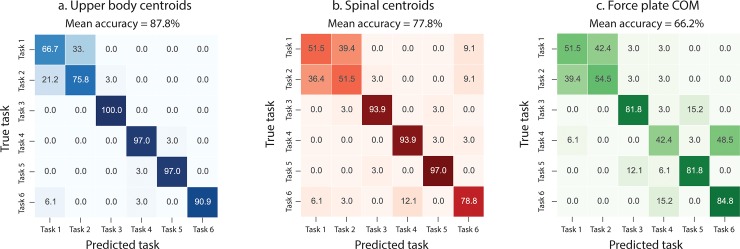
Confusion matrix showing the percent accuracy outcome of condition classification using a) MK2 centroids of the upper body, b) MK2 spinal centroids, and c) KFP center of pressure variance.

**Fig 8:**
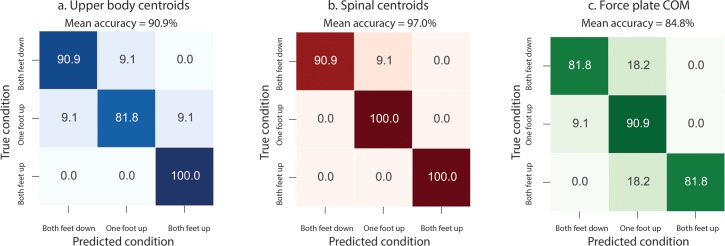
Confusion matrix showing the percent accuracy outcome of task classification using a) MK2 centroids of the upper body, b) MK2 spinal centroids, and c) KFP center of pressure variance.

Using the logistic regression classification method detailed previously, we input data from the MK2 to see how well we could classify the postural condition under which each mBBS task was performed. Performances are evaluated by mean of categorical accuracy. In order to represent a realist use-case of an unknown subject using the system for the first time, the evaluation is done with a leave-one-subject-out cross-validation procedure. Each subject is alternatively held out of the total training set, and used as a test subject. We repeated this procedure for each of our twelve subjects and the average accuracy is reported. The specific results of classifier performance are presented in Fig 8. The values in these confusion matrices represent the probability of our algorithm to correctly identify each condition. When analyzing all 16 of the selected MK2 upper-body centroids, average accuracy across all conditions was 90.9% (Fig 7A). We ran another analysis to find out how accurately our classifier could classify conditions using the KFP output, but this revealed an average accuracy of only 84.8% (Fig 7C). Furthermore, we found that when we used the classifier on the data from only the four spinal centroids, our classifier’s average accuracy increases to 97% (Fig 7B). This means that our classifier is 97% accurate when identifying postural motion using only the centroids that define the trunk. Indeed, 4 of the 6 tasks were performed while the subject had their arms crossed over their chests. Therefore, centroid corresponding to the arms and shoulders were not providing additional information. Moreover, the MK2 has difficulties in reliably locating upper limb joint centroids in this position, introducing more noise than information into the system when the arms are crossed.

### Classification of tasks from MK2 and KFP data

Our classifier also proved quite accurate at determining which task was being performed based upon MK2 and KFP data from all 12 subjects. Average accuracy of task classification from upper body MK2 and KFP COP data was 87.8% and 66.2%, respectively. Our classifier was most accurate at identifying each task when it used the 16-centroid data set from the MK2 (Fig 3B). Classifier performance was less accurate for the first two tasks, with both tasks only reaching 66.7% and 75.8% accuracy, respectively (Fig 8A). This is an expected result, considering the first and second task only differ in whether the subject’s eyes are open or closed. The remaining four tasks were kinematically unique from one another, allowing for much more robust classification. The KFP on the other hand, was far less accurate at discriminating the individual tasks. The force plate data did not allow for reliable classification of task 4, which was identified correctly only 42.4% of the time (Fig 8C). As before, we also evaluated the spinal centroids (Fig 3C) that carry the most information pertaining to postural stability in the seated position. The collective average accuracy of the prediction based on spinal centroids is 77.8% (Fig 8B). While it is advantageous to look only at the spine centroids when differentiating conditions, data from the 16 centroids of the upper body proved more helpful in distinguishing kinematically unique tasks.

### Accuracy of the KFP and MK2 as sample size increases

**Fig 9:**
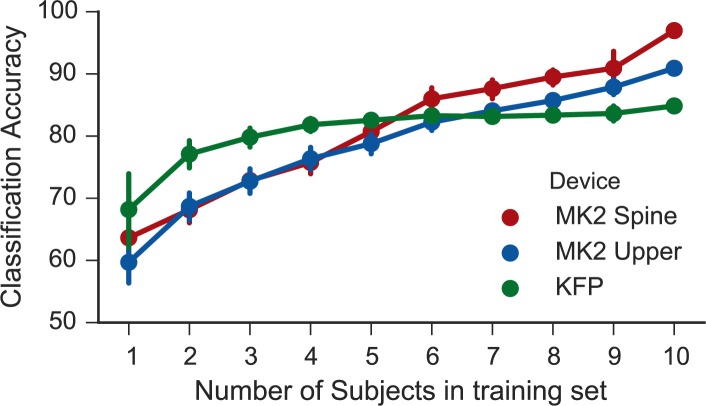
Accuracy of condition classification as number of subjects used to train the model increases.

As is typically the case, when using machine-learning concepts to analyze kinematic data, there is reason to believe that classification accuracy will increase as the size of the tested population increases. While this maxim holds for the MK2 data, the KFP shows no such trend (Fig 9). The KFP prediction accuracy plateaus after three to four subjects, whereas the MK2 classification accuracy continues to grow as the sample size increases. This can be explained by the fact that the KFP only provides data from a lower dimensional space (one 2D centroid compared with sixteen 3D centroids from the MK2), which limits the power of the classification technique when using data from the KFP. We predict that as a classification algorithm is used more and more frequently in clinical practice, its detection abilities will become more and more precise.

## Discussion

The MK2 is leading the field of affordable, whole-body markerless motion capture technology that is appropriate for home use. Several studies have utilized the MK1 or the MK2 for balance training and assessment and found reliability between both high-end motion capture and clinical scales [40–43], establishing that data from the MK1 and the MK2 is viable for such an approach. Here, we have proven that the MK2 can also make inferences about an individual’s postural stability. Building on this initial work, we will create a platform that can perform computerized balance assessments to identify individuals who are at risk of falling. Stratification of fall risk is related to a decrease in falls, and subsequently, a decrease in fall-related injuries [44]. An automated and computerized fall risk assessment that can be completed safely in a home environment will significantly aid attempts to stratify each user’s fall risk, and devote resources appropriately to fall prevention. This work is a necessary first step in developing such a platform.

While the Kinect is known to have high agreement with research-grade, markered motion-capture systems, its reliability as compared to a force plate has not been investigated as thoroughly. High-end force platforms that can perform detailed posturography are accurate, research-grade measuring tools in biomechanical studies of balance and postural stability [6,45]. Our study benchmarked the MK2 against the KFP, and found that the MK2 spinal centroid data showed a high level of agreement with the COP centroid from the KFP (Table 2). Previous work has also shown strong agreement between the MK2 and force-plate measurements of postural sway [41], and our study confirms these findings. Unlike research-grade devices such as the KFP, if is a financially viable and realistic option to deploy commodity devices such as the MK2 into home and clinical environments to assist in the stratification of fall-risk and objectively assess response to therapy. However, to date, no groups have developed and shared an analytics platform that can use data collected from the MK2 to accurately classify postural stability during task performance. This study presents an innovative methodology for meeting this need in the rehabilitation community.

We have determined that the data recorded by the MK2 is sufficient to classify three distinct states of postural stability in a healthy individual performing the mBBS. Previous studies that have attempted to use the MK 1 or 2 to quantify balance have shown encouraging results, but have not presented a methodology for analytics that could eventually allow for unsupervised data collection and classification. The goal of this work was to develop, demonstrate and release software that is capable of fulfilling these goals. In this study, we demonstrated a machine-learning algorithm with the ability to successfully differentiate between three distinct states of postural stability in a seated position with up to 97% accuracy based upon raw data collected by the MK2. The modified balance assessment that we developed allows for data collection to occur in a seated position, and to only involve assessment items that did not put the subject at any risk of falling, which is a concern for existing fall assessments such as the BBS. Because of the safety and speed with which our protocol may be performed, we think our assessment could feasibly be performed in the home, making it a strong candidate for many telemedicine applications.

Furthermore, our data analysis protocols revealed that we could use data from the MK2 to robustly determine which of the six tasks were being performed. Our classifier showed 87.8% accuracy in predicting which of the tasks were being performed, and was only largely inaccurate in differentiating between the first two tasks: sitting with eyes open (task one) and sitting with eyes closed (task two). These analyses are important to pursue, because they provide a framework for creating robust protocols for future home-use applications, by allowing researchers to perform remote evaluation of data quality for each task. For instance, if ability to classify the difference between task 3 and 4 drops dramatically in a subject’s balance assessment data, a researcher would be able to infer that something is going wrong with the data collection protocol: perhaps the MK2 sensor is performing poorly, or the task is being performed incorrectly. This would be an indication that researchers should check in with a research subject, rather than continue to collect low-quality data and only know.

### Limitations and considerations

The main limitation of our study was the use of healthy subjects. While this is a necessary step in the benchmarking and machine learning process, we are unable to draw any conclusions about how the classifier may perform in patient populations. Another factor for consideration is that our experimental data was collected under controlled conditions: all data was collected in a lab environment with controlled light, background and seating conditions. Since certain environmental conditions can degrade the signal-to-noise ratio of the raw Kinect data, we must also conduct experiments to measure the impact that variable environmental conditions can have on data quality, and therefore classification performance. In-home studies would have to be conducted in order to predict the extent and effect of the noise in question.

### Conclusions and future directions

We have presented novel methodology that can repurpose a widely available consumer hardware product with a custom software platform that has the potential to detect individuals who may be at risk of falling. From a telemedicine perspective, establishing our motion capture and analysis algorithms in a healthy population is an exciting first step into a larger sphere of innovative possibilities:

- Remote physical therapy assessments
- Continuous kinematic feedback to individual users during home exercise completion
- Alerts sent to the therapist if a patient is losing function between clinic visits
- An improved ability to conduct longitudinal clinical trials with multiple, quantitative observations taken in the home environment rather than requiring multiple clinical visits.

In order to come closer to these goals, a future focus of this work will be centered on developing a user interface that is simple and usable, such that it will allow quick and easy data capture—even when it is being used by individuals with little to no previous experience with using computers. The potential of this work cannot be understated: the MK2 is a device that can be rapidly and cost-effectively deployed to homes and clinics globally. The methods presented here for classifying motor behavior can be expanded for multiple conditions, allowing individuals with motor impairments to be monitored more closely and treated more safely and effectively.

Another future direction for this work is to begin data collection on a large number of individuals who are at risk of falling, including, but not limited to, elderly individuals, people with spinal cord injury, people with Parkinson’s Disease and stroke survivors. Our protocol and resulting data will allow us to test the classifier in different clinical and non-clinical populations, and allow us to correlate our MK2 data with clinical gold standards such as the Berg Balance Scale.

Finally, our long-term vision for this work is to truly revolutionize the nature of clinical outcome assessment itself: currently, clinical researchers must select from a myriad of clinical outcome measures, each with varying degrees of applicability, sensitivity, and validation for different study populations. Automated visual analysis would extract more salient information regarding the individual’s natural function in his or her own home environment. This approach, which has *never been implemented before*, has the potential to replace subjectively assessed clinical testing protocols. This would drastically improve data interoperability across different clinical trials, achieving one of the main goals of the National Institutes of Health’s ‘Common Data Elements’ initiative.
